# Real-World Application of PD-1 Inhibitors in High-Grade Glioma: A Case Series

**DOI:** 10.3390/cancers18193122

**Published:** 2026-09-26

**Authors:** Yin Ren, Xiaopeng Guo, Hao Xing, Xiaoman Kang, Tingyu Liang, Yixin Shi, Bohan Yao, Wenbin Ma, Yu Wang

**Affiliations:** Department of Neurosurgery, Peking Union Medical College Hospital, Chinese Academy of Medical Sciences & Peking Union Medical College, Beijing 100730, China; b2024001217@student.pumc.edu.cn (Y.R.); guoxiaopeng@pumch.cn (X.G.);

**Keywords:** high-grade glioma, PD-1 blockade, combination therapy, VEGFR-targeted therapy, neoadjuvant anti-PD-1 therapy

## Abstract

**Background/Objectives**: Patients with high-grade glioma (HGG) have a poor prognosis despite maximal safe resection, radiotherapy, and chemotherapy, partly because of the immunosuppressive tumor microenvironment. Unfortunately, all phase III trials of anti-PD-1 therapy to date have failed to yield positive results in HGGs. Recent early-phase trials have identified promising strategies involving immune checkpoint inhibitors. Here, we share our 8-year experience with anti-PD-1 therapy in patients with HGG in a real-world setting. **Methods**: All patients with HGG who received PD-1 inhibitor-based therapy at our center between 2018 and 2026 were included. Medical records were reviewed longitudinally for each patient, and the latest follow-up data were collected. Patients lost to follow-up were excluded. **Results**: Four regimens involving anti-PD-1 treatment were identified in 22 patients with HGG (newly diagnosed = 11, recurrent = 11): (1) VEGFR-targeted therapy plus PD-1 inhibition (9 recurrent HGGs), (2) the “2-THE-TOP” regimen (tumor-treating fields plus temozolomide and PD-1 inhibitor therapy) (5 newly diagnosed HGGs), (3) anti-PD-1 monotherapy (2 recurrent and 2 newly diagnosed HGGs), and (4) neoadjuvant plus adjuvant anti-PD-1 therapy (4 newly diagnosed HGGs). Median overall survival for these four regimens was 32.7, 20.3, 28.6, and 31.8 months, respectively. No grade 3–4 treatment-related adverse events were recorded. **Conclusions**: Our preliminary observations suggest that PD-1 blockade may remain a viable option for patients with HGG when combined with specific therapies, administered with careful timing, and applied in selected patients.

## Simple Summary

High-grade gliomas (HGGs) represent a group of aggressive primary brain tumors with poor prognosis despite current standard-of-care therapies. Although PD-1 inhibitors have revolutionized immunotherapy in multiple solid malignancies, all phase III trials in HGGs to date have yielded negative results. Recently, however, encouraging signals have emerged from phase I/II trials employing multi-modal strategies involving PD-1 inhibition, providing inspiration to our clinical practice. In this cohort, we retrospectively analyzed 22 patients with HGG who were treated with PD-1 blockade at our institution over the past decade, and identified four treatment strategies with satisfactory outcomes: combination of a PD-1 inhibitor and an anti-angiogenic agent, integration of a PD-1 inhibitor with tumor-treating fields and temozolomide, anti-PD-1 monotherapy, and neoadjuvant-plus-adjuvant PD-1 inhibition. This real-world study suggests that anti-PD-1 therapy may benefit patients with HGG when treatment regimens are optimized, treatment timing is refined, and patients are selected based on biomarkers. These findings may help guide clinical decision-making.

## 1. Introduction

High-grade gliomas (HGGs), particularly glioblastoma (GBM), are the most common primary malignant brain tumors in adults [1,2,3]. Despite the standard-of-care treatment regimen, encompassing maximal safe resection, concurrent chemoradiotherapy, adjuvant chemotherapy with temozolomide (TMZ), and tumor-treating fields (TTFields) therapy, the prognosis for these patients remains exceedingly poor, with a median overall survival (OS) of less than 2 years and a 5-year survival rate of <10% [1,4,5]. In addition, almost all patients experience recurrence [4,6,7,8,9]. The dismal prognosis is partly attributable to the infiltrative growth pattern of malignant tumor cells and the blood–brain barrier, which impedes drug delivery, and, more importantly, to the immunosuppressive tumor microenvironment (TME) [7,10,11].

To date, major phase III clinical trials evaluating PD-1 inhibitor usage in HGGs have failed to improve patient survival [12,13,14,15]. The failure of these trials suggests that substituting or adding a single immune checkpoint inhibitor might be insufficient to overcome the immunosuppressive TME of HGGs [7,8]. Recently, however, encouraging signals have emerged from phase I/II trials employing multimodal combination strategies, including PD-1 inhibition. Synergistic efficacy has been implied with the concurrent use of anti-PD-1 therapy with anti-angiogenic agents [16], with neoadjuvant PD-1 blockade prior to surgery [17], and with the “2-THE-TOP” regimen integrating PD-1 inhibitors with TTFields and TMZ [18]. Survival benefits were also observed when using anti-PD-1 monotherapy in HGGs harboring high tumor mutation burden (TMB), mismatch repair deficiency (dMMR), POLE mutation, and DNA replication repair deficiency [19,20,21,22]. Since the above findings remain preliminary, there is a critical need to expand the patient cohort and validate these signals to assist in identifying the optimal use of PD-1 blockade in HGGs.

As a high-volume center for diffuse glioma treatment, our institution has accumulated over a decade of experience in applying PD-1 blockade therapy, either as monotherapy or in combination with other treatment modalities, in HGGs. We retrospectively reviewed the medical records of patients with HGG and summarized the clinical outcomes of four major PD-1 inhibitor-based treatment regimens: VEGFR-targeted therapy plus PD-1 blockade, the “2-THE-TOP” regimen, adjuvant PD-1 blockade monotherapy (in selected patients), and neoadjuvant plus adjuvant PD-1 blockade therapy.

## 2. Materials and Methods

### 2.1. Study Participants and Ethics

Patients with WHO grade 3–4 gliomas, either IDH-wildtype or IDH-mutant, who received PD-1 blockade therapy (camrelizumab or pembrolizumab) and had serial follow-up at the Department of Neurosurgery, Peking Union Medical College Hospital, between November 2018 and July 2026 were included. Patients with less than 3 months of follow-up data were excluded.

This study was approved by the Ethics Committee of Peking Union Medical College Hospital (HS-2522) and was conducted in accordance with the Declaration of Helsinki. All patients provided written informed consent.

### 2.2. Data Collection

Patient neuroimaging data and clinicopathological features, including age at diagnosis, sex, tumor location, date of surgery, pathological diagnosis, and molecular profiling, were collected for each patient. Treatment regimens involving PD-1 blockade were recorded, and all follow-up records were reviewed. Baseline MR images were evaluated using the MRI VASARI lexicon v2.0 [23], and the majority of patients exhibited similar preoperative imaging features (Table S1). During follow-up, radiological response was assessed using the Immunotherapy Response Assessment in Neuro-Oncology (iRANO) criteria [24].

### 2.3. Treatment Patterns with PD-1 Inhibitor

Four major treatment patterns were identified.

*VEGFR-targeted plus PD-1 blockade therapy for recurrent patients:* All patients received at least one cycle of combination therapy of a PD-1 inhibitor (pembrolizumab 100–200 mg every 4 weeks) and an anti-angiogenic agent (bevacizumab 3–5 mg/kg every 3–4 weeks).

*The “2-THE-TOP” regimen for newly diagnosed patients:* Patients received combination treatment consisting of TTFields, PD-1 blockade (pembrolizumab 100–200 mg every 4 weeks), and TMZ (150–200 mg/m^2^ daily for 5 days every 28 days) after surgery.

*PD-1 blockade monotherapy for newly diagnosed and recurrent patients:* For newly diagnosed patients, a PD-1 inhibitor (pembrolizumab 100–200 mg every 3–4 weeks) was administered postoperatively in addition to TMZ-based chemoradiation and chemotherapy; for recurrent patients, PD-1 inhibitor therapy was prescribed upon progression with best physician-chosen therapies. Molecular testing was performed in these four patients, allowing for the determination of mismatch repair status or tumor mutational burden.

*Neoadjuvant plus adjuvant PD-1 blockade therapy for newly diagnosed patients:* All patients received a single dose of a PD-1 inhibitor (camrelizumab 200 mg) within 2 weeks prior to surgery, followed by adjuvant postoperative camrelizumab (200 mg) every 2 weeks in addition to standard therapy.

### 2.4. Statistical Analysis

Descriptive statistics were used to summarize clinical features. Progression-free survival (PFS) and OS were calculated for each patient. For patients with recurrent gliomas, PFS was calculated from the time of first radiological evidence of progression to the next radiological progression or all-cause death, and OS was calculated from initial surgery to all-cause death. For patients with newly diagnosed gliomas, PFS was calculated from initial surgery to the first radiological evidence of progression or death, and OS was calculated from initial surgery to all-cause death. For PFS, patients without documented progression or death were censored at the last follow-up. For OS, patients who were still alive were censored at the last follow-up. All-cause death was treated as an event for both endpoints.

## 3. Results

### 3.1. Study Cohort

A total of 22 patients, 13 males and 9 females, with a mean age of 52 years, were enrolled. Gliomas included 20 GBMs and 2 grade 4 astrocytomas; of these 22 patients, 11 were newly diagnosed and 11 had recurrent disease (Table 1). At the last follow-up, the censoring rates for PFS and OS were 0% and 0% in the VEGFR-targeted plus PD-1 blockade group, 0% and 20.0% in the “2-THE-TOP” group, 75.0% and 100.0% in the PD-1 blockade monotherapy group, and 25% and 25.0% in the neoadjuvant plus adjuvant group, respectively (Figure 1).

Detailed treatments and responses of each patient throughout the treatment course are illustrated in Figure 1. At the last follow-up, 16 patients had died and 6 were still alive. No grade 3–4 adverse events were recorded (Table 1).

### 3.2. Pattern 1: VEGFR-Targeted Plus PD-1 Blockade Therapy

Nine patients with recurrent GBM received combination therapy with VEGFR-targeted agents and PD-1 inhibitors. For these patients, PFS was 32.3, 26.1, 4.1, 13.7, 21.3, 10.2, 4.2, 11.2, and 9.3 months, with a corresponding OS of 48.8, 47.5, 9.9, 32.7, 48.6, 35.5, 21.6, 26.1, and 24.9 months (Table 1). Median PFS was 11.2 months (range, 4.1 to 32.3 months), and median OS was 32.7 months (range, 9.9 to 48.8 months).

A representative case is presented in Figure 2. This was a 56-year-old male who presented with a space-occupying lesion in the right temporal lobe. He underwent surgical resection on 8 November 2018, and was pathologically diagnosed with GBM. He subsequently received concurrent chemoradiotherapy with TMZ from 28 December 2018 to 4 February 2019, followed by eight cycles of adjuvant TMZ chemotherapy until August 2019. Tumor progression was documented on 19 August 2019. The patient then received a combination regimen of bevacizumab plus pembrolizumab from 27 May 2020 to 25 October 2022. A second tumor progression was observed on 24 May 2022, and the patient succumbed to the disease on 3 December 2022. The PFS and OS for this patient were 32.3 months and 48.8 months, respectively.

### 3.3. Pattern 2: The “2-THE-TOP” Regimen

Five patients with newly diagnosed grade 4 gliomas (4 GBMs and 1 astrocytoma) received the “2-THE-TOP” regimen following concurrent chemoradiotherapy with TMZ. Among these patients, four had died, with PFS of 16.8, 10.2, 3.1, and 11.6 months, respectively, and OS of 25.1, 15.8, 11.2, and 20.3 months. The remaining patient was still alive at the last follow-up, with a PFS and OS of 14.1 and 22.9 months (Table 1). Median PFS was 11.6 months (range, 3.1 to 16.8 months), and median OS was 20.3 months (range, 11.2 to 25.1 months).

A representative case is presented in Figure 3. This was a 42-year-old male who presented with a space-occupying lesion in the right frontal lobe. He underwent surgical resection on September 11, 2024, and was pathologically diagnosed with astrocytoma, IDH-mutant, WHO grade 4. He initiated TTFields therapy on 16 October 2024, and received concurrent chemoradiotherapy with TMZ from 30 October 2024 to 6 December 2024. Subsequently, the patient received 10 cycles of the “2-THE-TOP” regimen from 13 March 2025 to 5 November 2025. Tumor progression was documented on 14 November 2025. On 15 November 2025, an Ommaya reservoir was implanted for intraventricular administration of cytarabine. The patient subsequently underwent CyberKnife radiosurgery for a lesion in the fourth ventricle on 15 December 2025, followed by one cycle of combination therapy of bevacizumab plus pembrolizumab on 30 December 2025. The patient was still alive at the last follow-up. The PFS for this patient was 14.1 months, and the OS to date was 22.9 months.

### 3.4. Pattern 3: Adjuvant Anti-PD-1 Monotherapy for Select Patients

Three patients with GBMs and one with grade-4 astrocytoma received adjuvant anti-PD-1 therapy. Molecular testing showed dMMR in two patients: one with recurrent GBM and one with newly diagnosed GBM. The other two patients harbored an ultra-high TMB score, with 608 mutations/Mb in one newly diagnosed GBM patient and 392 mutations/Mb in one patient with recurrent grade 4 astrocytoma. All four patients were alive at the last follow-up. One patient experienced disease progression, with a PFS of 12.2 months and an OS of 39.4 months, whereas the remaining three patients remained progression-free and alive, with PFS censored at 14.9, 18.1, and 29.2 months and OS censored at 27.9, 18.1, and 29.2 months, respectively (Table 1). Median PFS was 16.5 months (range, 12.2 to >29.2 months; three patients censored), and median OS was 28.6 months (range, 18.1 to >39.4 months; all patients censored).

A representative case is presented in Figure 4. This was a 50-year-old male with a left temporoparietal GBM with dMMR. He underwent surgical resection on 15 April 2023, followed by concurrent chemoradiotherapy with TMZ, and subsequently received 14 cycles of adjuvant TMZ from 15 November 2023 to 15 January 2025. Tumor progression was documented on 18 December 2024, and a second surgery was performed on January 13, 2025. Then he received 15 cycles of PD-1 blockade therapy from 15 January 2025 to 15 December 2025. On 24 December 2025, the patient experienced a second recurrence with evidence of ventricular dissemination. The PFS for this patient was 12.2 months after receiving PD-1 inhibitor therapy. In February 2026, the patient was started on a combination of everolimus, trametinib, and bevacizumab; however, disease progression was documented again on 22 June 2026, leading to discontinuation of this regimen. The patient was still alive at the last follow-up, with an OS of 39.4 months. Figure 4 presents the clinical course of this patient starting from the first tumor progression on 18 December 2024.

### 3.5. Pattern 4: Neoadjuvant Plus Adjuvant PD-1 Blockade Therapy

Four patients with newly diagnosed GBMs were treated with preoperative (neoadjuvant) and postoperative (adjuvant) PD-1 blockade therapy, in addition to concurrent chemoradiotherapy and adjuvant TMZ. Among these four patients, three had died, with a PFS of 30.3, 6.5, and 11.1 months and an OS of 39.7, 23.9, and 12.7 months. The remaining patient was still alive without disease progression at the last follow-up, with a PFS and an OS of 57.9 months (Table 1). Median PFS was 20.7 months (range, 6.5 to >57.9 months; one patient censored), and median OS was 31.8 months (range, 12.7 to >57.9 months; one patient censored).

A representative case is presented in Figure 5. This was a 68-year-old male with a GBM in the left frontal lobe. He received neoadjuvant camrelizumab on 30 September 2021, followed by surgical resection on 8 October 2021. He subsequently underwent concurrent chemoradiotherapy with TMZ from 18 November 2021 to 29 December 2021, and received 3 cycles of adjuvant TMZ chemotherapy from 7 February 2022 to 4 April 2022. In addition, the patient received 18 cycles of camrelizumab immunotherapy from 15 October 2021 to 22 September 2022. Contrast-enhanced MRI from 11 October 2021 to 6 July 2022, revealed an enhancing area surrounding the resection cavity, which was considered most likely consistent with pseudoprogression at that time; thus, no specific intervention was administered, and regular surveillance imaging was continued. On 22 May 2024, follow-up contrast-enhanced MRI demonstrated that the perilesional enhancement had essentially resolved. The patient was still alive without progression at the last follow-up, with PFS and OS of 57.9 months.

## 4. Discussion

We reviewed the treatment courses and outcomes of 22 patients who received anti-PD-1 therapy at our institution during the past decade and identified four treatment patterns that appeared to yield favorable outcomes. Despite the negative results of phase III trials of PD-1 inhibition in GBM [12,13,14,15], our real-world experience with PD-1 blockade was encouraged by recent phase I/II studies with relatively satisfactory results, when anti-PD-1 therapy was applied together with bevacizumab, with TTFields plus TMZ or when its timing of administration was optimized [17,18,22,25,26]. In addition, patients with specific molecular features, including dMMR [19,21], *POLE* mutations [20], and high TMB [22], were more likely to derive clinical benefits from PD-1 inhibition.

For recurrent GBMs, a recognized standard of care remains elusive [27], with bevacizumab, a VEGFR-targeting agent, standing as an accepted therapeutic option. Although no OS benefit was observed, bevacizumab has been shown to more than double the median PFS, extending it from 1.5 months to 4.2 months [28]. In head-to-head phase III comparisons, PD-1 inhibitors have failed to improve OS compared with bevacizumab [12]. Encouragingly, a number of recently published early-phase clinical trials and case reports have pointed toward a potential synergistic effect when bevacizumab is added to PD-1 blockade [16,29,30,31,32,33,34]. Although conflicting evidence remains [35], the majority of studies suggested that the combination of bevacizumab and a PD-1 inhibitor conferred a survival advantage in patients with recurrent HGG. Bevacizumab synergizes with PD-1 blockade by reversing VEGF-mediated immunosuppression, including impaired dendritic cell maturation, enhanced regulatory T cell proliferation, and suppressed T cell function, while simultaneously promoting tumor vascular normalization and thereby increasing effector T cell infiltration into the tumor [16,36,37,38]. This benefit may be attributable to molecularly guided patient selection and optimized bevacizumab dosing [31].

TTFields therapy suppresses tumor growth through inhibition of mitotic spindle formation [39,40]. When applied during adjuvant TMZ chemotherapy, TTFields therapy has been shown to extend median OS to 20.9 months [4]. Beyond this direct cytostatic effect, TTFields therapy also engages the cGAS-STING pathway, potentiating antitumor immunity and creating a rational basis for combination with immune checkpoint blockade [41,42,43]. In line with this mechanistic rationale, a recent phase II trial reported encouraging efficacy with the combination of TTFields, a PD-1 inhibitor, and TMZ [18], and a phase III study is currently underway (NCT06556563). By incorporating this strategy into our clinical practice, we have likewise observed encouraging OS and PFS in patients with HGG.

PD-1 monotherapy conferred limited survival benefit in recurrent HGGs when applied to unselected patients [12,13,15,35,44], with little consideration of patients’ molecular profiles. It is plausible that by refining patient selection, specifically by identifying those more likely to respond to PD-1 blockade, the therapeutic efficacy of immunotherapy could be improved [19,20,21,22,45,46]. In our series of four patients, two harbored high TMB and two exhibited dMMR. These patients achieved favorable outcomes with PD-1 inhibitor monotherapy. This observation supported an individualized, biomarker-guided approach when considering PD-1 inhibition in patients with HGG.

The neoadjuvant administration of PD-1 inhibitors has drawn increasing attention in recent years and has yielded breakthroughs in various solid tumors [47,48,49]. Within the glioma field, three published studies have suggested that this treatment paradigm may be effective [17,25,50]. The underlying mechanism involves the relief of T-cell immunosuppression in the presence of tumor antigens, thereby promoting clonal expansion and functional activation of tumor-specific T cells. This process, in turn, leads to the upregulation of interferon-γ-related genes and the downregulation of cell cycle and cancer proliferation-related genes within the tumor microenvironment, ultimately enhancing both local and systemic antitumor immune responses [17,25,50,51]. In light of these results, we initiated a single-arm clinical trial based on this neoadjuvant strategy at our center (NCT04583020). To date, four patients have been enrolled, and preliminary results have shown encouraging OS and PFS.

Our study has limitations. First, as a single-center retrospective study, the sample size was relatively small, leading to potential bias across the various treatment groups. Given the retrospective and descriptive nature of this study, we presented the treatment course and responses of each patient to facilitate interpretation of our findings. Moreover, PD-1/PD-L1 interaction, PD-L1 expression, and CD8+ T cell infiltration were not routinely assessed in this study; instead, the TMB and dMMR status of tumors were assessed in patients before anti-PD-1 monotherapy was initiated. Future studies incorporating assessment of the immune microenvironment might further refine patient selection for PD-1 blockade.

## 5. Conclusions

In summary, we report our experience with 22 patients treated with anti-PD-1 therapy and propose that the efficacy of PD-1 blockade may be improved by optimizing combination regimens, refining patient selection, and adjusting the timing of administration. Given the difficulty of obtaining large-scale phase III results, this study provides a valuable reference for the clinical application of PD-1 inhibitors in HGGs. These findings warrant further validation in larger cohorts.

## Figures and Tables

**Figure 1:**
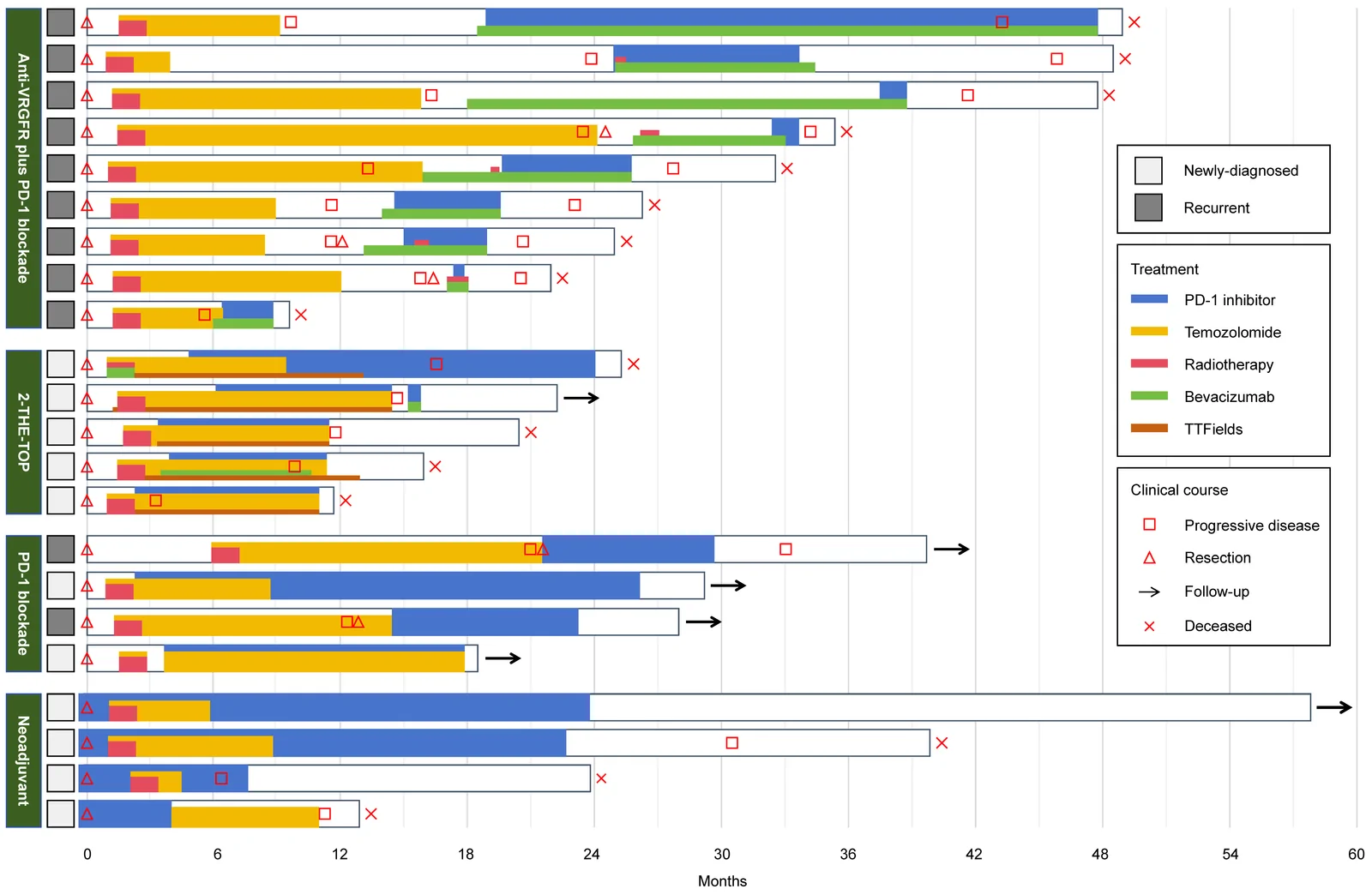
Clinical course of all included cases with high-grade gliomas.

**Figure 2:**
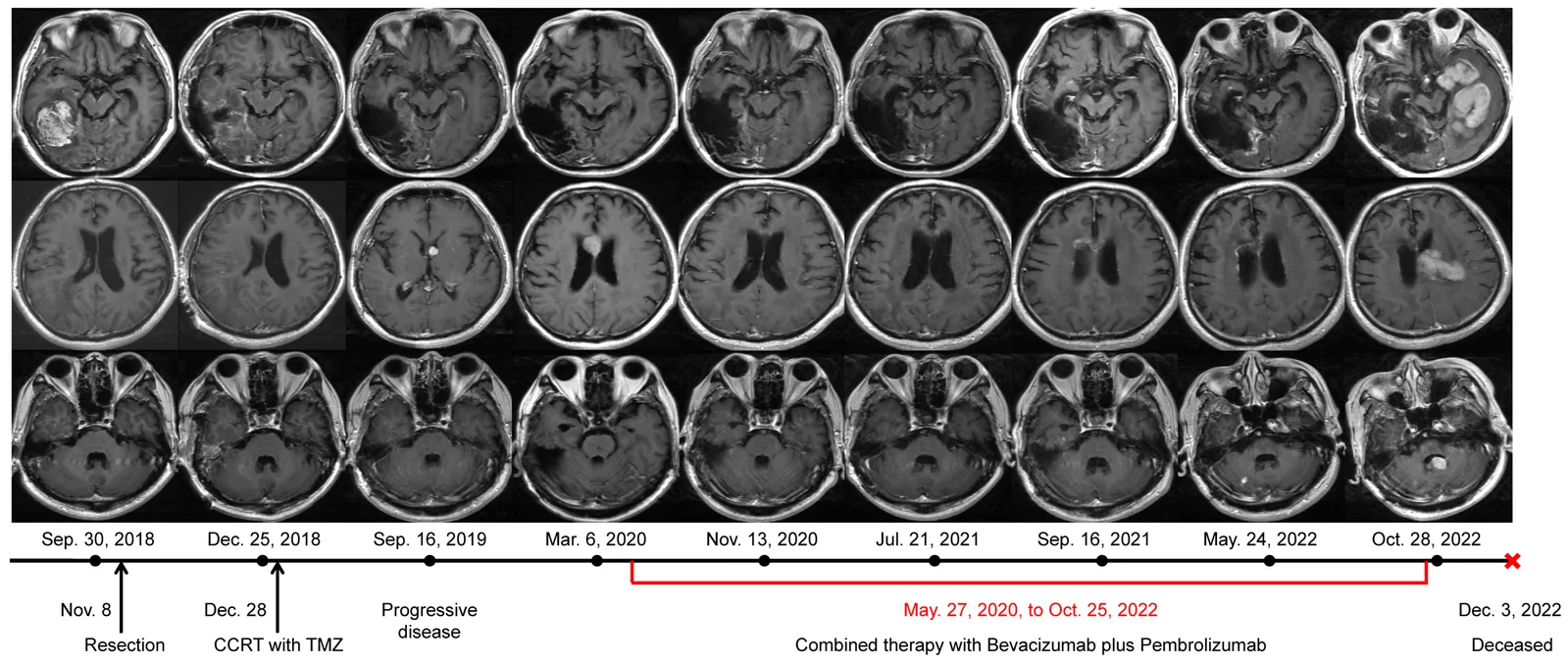
This 56-year-old male with a right temporal lobe lesion underwent surgical resection and was diagnosed with GBM, IDH-wildtype. He received concurrent chemoradiotherapy with TMZ followed by eight cycles of adjuvant TMZ before disease progression. He subsequently received bevacizumab plus pembrolizumab for 29 months, achieving a PFS of 32.3 months and OS of 48.8 months.

**Figure 3:**
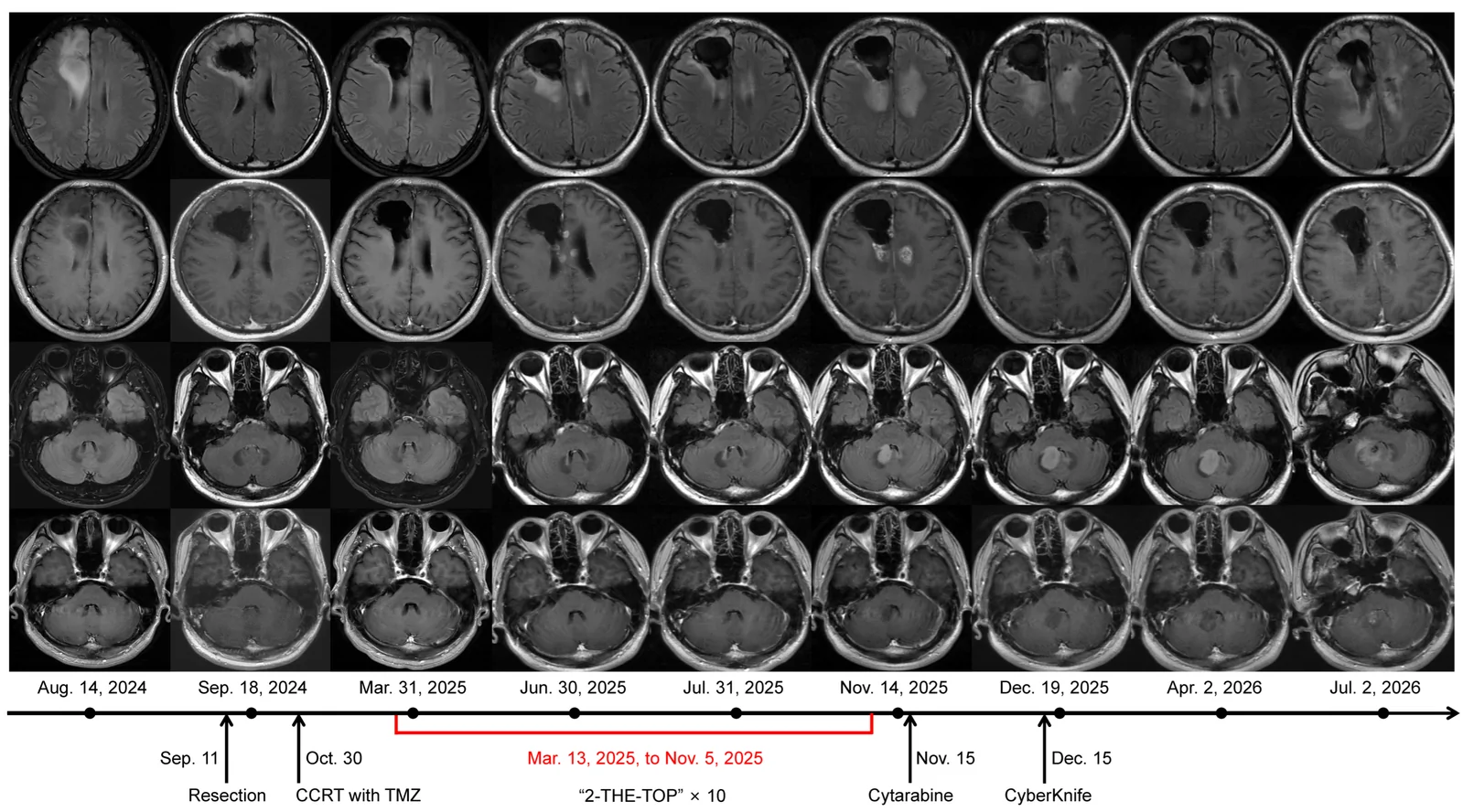
This 42-year-old male patient with a right frontal grade-4 astrocytoma received concurrent chemoradiotherapy with TMZ, followed by 10 cycles of the “2-THE-TOP” regimen. The patient remained alive at the last follow-up, with a PFS of 14.1 months and OS of >22.9 months.

**Figure 4:**
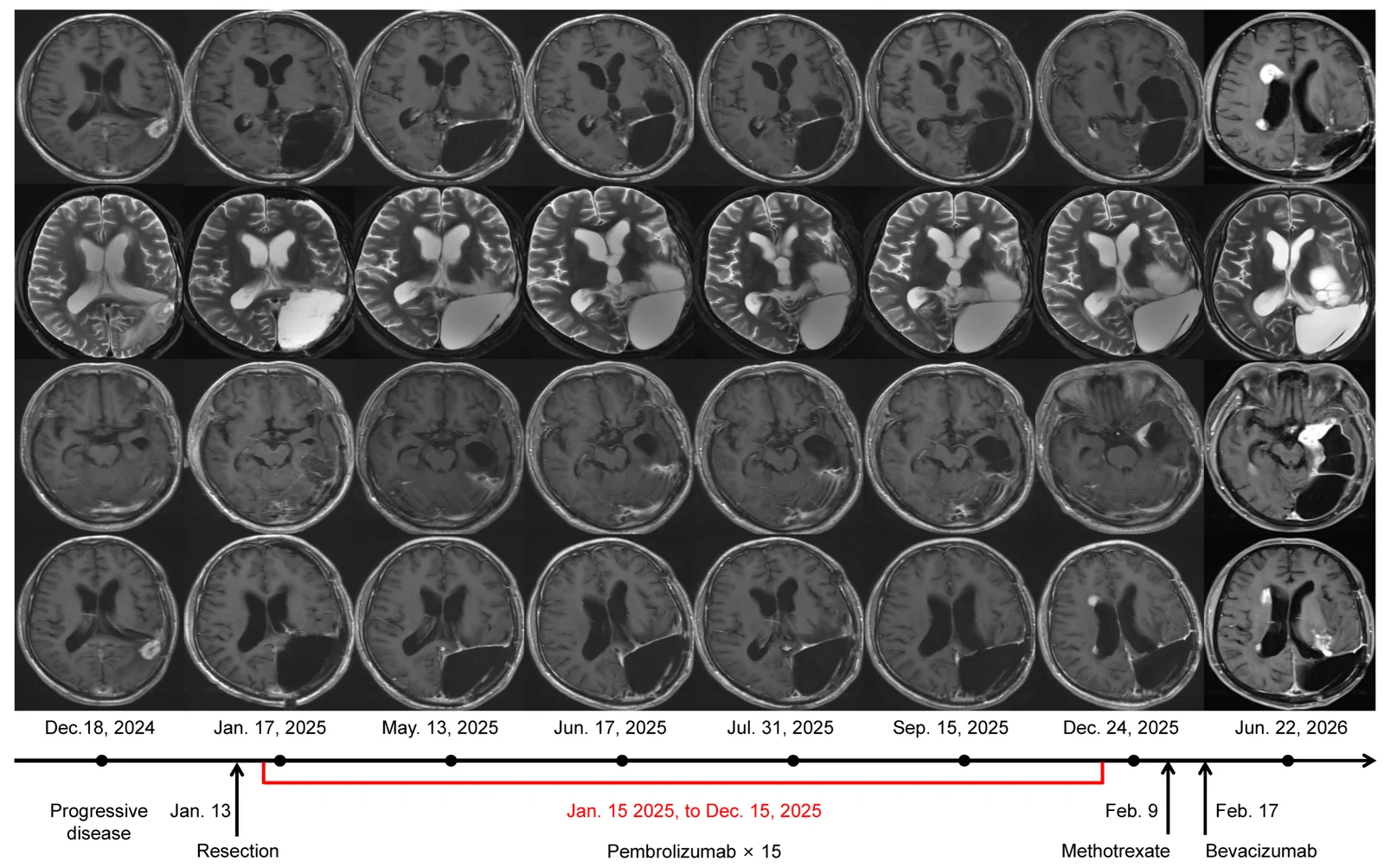
This was a 50-year-old man who presented with a left temporoparietal lesion. He underwent initial resection, followed by concurrent chemoradiotherapy with TMZ and 14 cycles of adjuvant TMZ before recurrence. He received a second surgery and another 15 cycles of PD-1 blockade until the second on December 2025. The patient remains alive at the last follow-up, with a PFS of 12.2 months after PD-1 therapy and an OS of 39.4 months.

**Figure 5:**
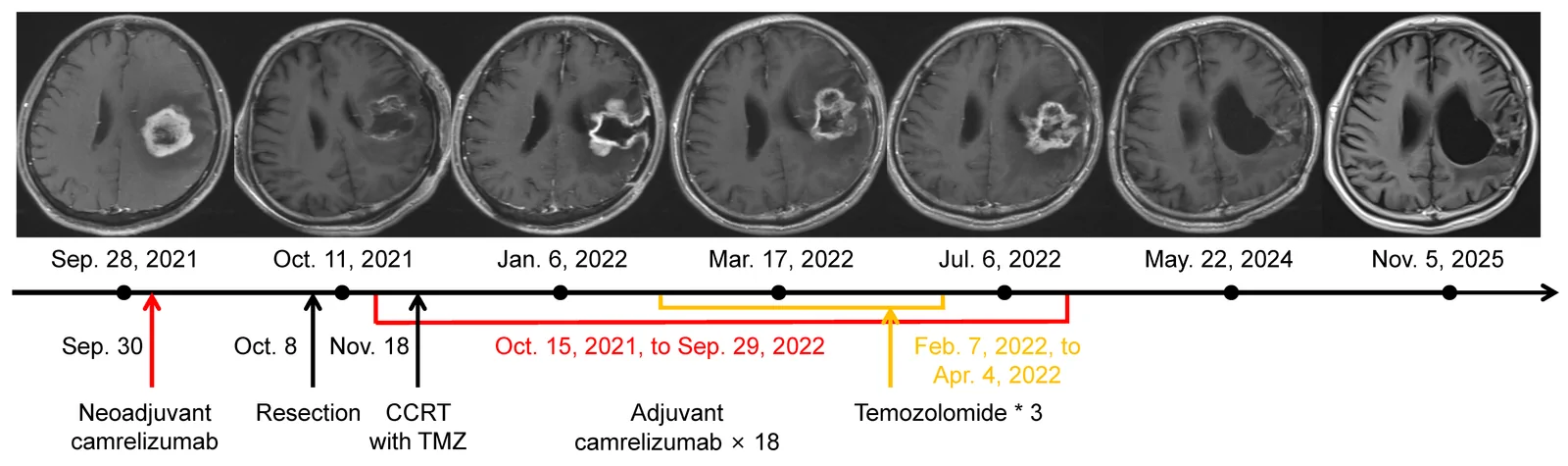
This 68-year-old male with a left frontal GBM received neoadjuvant camrelizumab 8 days before surgery and concurrent chemoradiotherapy with TMZ, 3 cycles of adjuvant TMZ, and 18 cycles of camrelizumab postoperatively. The patient remains alive without progression, with PFS and OS of 57.9 months.

**Table 1:** Baseline Characteristics of All Participants.

Treatment	Age, Years	Sex	Tumor Location	Tumor Subtype	WHO Grade	Status	PFS, Months	OS, Months	Adverse Event
VEGFR-targeted plus PD-1 blockade therapy	56	Male	Right temporal	Glioblastoma	4	Recurrent	32.3	48.8	2
	53	Female	Left temporooccipital	Glioblastoma	4	Recurrent	26.1	47.5	1
	29	Male	Right temporoparietal	Glioblastoma	4	Recurrent	4.1	9.9	1
	71	Male	Left parietooccipital	Glioblastoma	4	Recurrent	13.7	32.7	2
	51	Male	Right temporal	Glioblastoma	4	Recurrent	21.3	48.6	2
	53	Female	Right frontotemporal	Glioblastoma	4	Recurrent	10.2	35.5	1
	51	Male	Right temporoinsular	Glioblastoma	4	Recurrent	4.2	21.6	None
	61	Male	Left frontal	Glioblastoma	4	Recurrent	11.2	26.1	1
	32	Male	Right temporoparietooccipital	Glioblastoma	4	Recurrent	9.3	24.9	2
The “2-THE-TOP” regimen	44	Female	Left frontal	Glioblastoma	4	ND	16.8	25.1	2
	45	Female	Left frontal	Glioblastoma	4	ND	10.2	15.8	1
	54	Female	Right frontal	Glioblastoma	4	ND	3.1	11.2	1
	63	Female	Left temporal	Glioblastoma	4	ND	11.6	20.3	2
	42	Male	Right frontal	Astrocytoma	4	ND	14.1	>22.9 *	None
Adjuvant PD-1 blockade monotherapy	50	Male	Left temporoparietal	Glioblastoma	4	Recurrent	12.2	>39.4 *	2
	35	Male	Left parieto-occipital	Glioblastoma	4	ND	>29.2 *	>29.2 *	2
	65	Female	Right frontal	Glioblastoma	4	ND	>18.1 *	>18.1 *	2
	38	Female	Left frontotemporal	Astrocytoma	4	Recurrent	>14.9 *	>27.9 *	1
Neoadjuvant plus adjuvant PD-1 blockade therapy	54	Male	Right temporal	Glioblastoma	4	ND	30.3	39.7	1
	70	Female	Left frontal	Glioblastoma	4	ND	6.5	23.9	2
	68	Male	Left frontal	Glioblastoma	4	ND	>57.9 *	>57.9 *	1
	58	Male	Left frontotemporoparietal	Glioblastoma	4	ND	11.1	12.7	2

OS was calculated starting from the day of initial surgery for all groups. PFS was calculated from initial surgery (newly diagnosed) or from first progression imaging (recurrent) to subsequent progression. * indicated that the survival endpoint was not reached. Abbreviation: ND = newly diagnosed.

## Data Availability

The raw data supporting the analysis of this article are available from the corresponding author upon reasonable request.

## Supplementary Materials

The following supporting information can be downloaded at: https://www.mdpi.com/article/10.3390/cancers18193122/s1, Table S1: Preoperative imaging assessment of all patients based on the VASARI 2.0 scoring system.

## Abbreviations

The following abbreviations are used in this manuscript:

HGG	high-grade glioma
GBM	glioblastoma
TMZ	temozolomide
TTFields	tumor-treating fields
OS	overall survival
TME	tumor microenvironment
TMB	tumor mutation burden
dMMR	mismatch repair deficiency
PFS	progression-free survival
