# Supplementary material for: Real-World Application of PD-1 Inhibitors in High-Grade Glioma: A Case Series
Source: Cancers (Basel). 2026 Sep 26;18(19):3122. doi: 10.3390/cancers18193122 (PMC13664033; doi:10.3390/cancers18193122)
Supplement: Supplementary file 1 [file cancers-18-03122-s001.zip › cancers-4548103-supplementary.pdf]

Table S1. Preoperative imaging assessment of all patients based on the VASARI 2.0 scoring system.

Treatment	Age, years	Sex	F4 Enhancement quality	F5 Enhanced area	F6 No-enhanced area	F7 Necrosis area	F14 Edema area	F17 Diffusion quality
VEGFR-targeted plus PD-1 blockade therapy	56	Male	3	3	2	2	2	3
	53	Female	3	4	1	1	1	2
	29	Male	3	2	2	3	1	2
	71	Male	3	3	1	2	2	1
	51	Male	3	3	2	2	2	3
	53	Female	3	3	1	1	2	2
	51	Male	3	3	2	2	2	3
	61	Male	3	4	1	1	2	2
	32	Male	3	3	1	3	1	1
The "2-THE-TOP" regimen	44	Female	3	3	2	2	1	1
	45	Female	3	3	2	2	1	2
	54	Female	2	1	6	1	1	2
	63	Female	2	2	2	1	3	3
	42	Male	1	1	6	1	1	2
Adjuvant PD-1 blockade monotherapy	50	Male	3	3	2	2	2	1
	35	Male	3	3	2	1	2	3
	65	Female	3	3	1	1	2	2
	38	Female	3	4	2	1	2	3
Neoadjuvant plus adjuvant PD-1 blockade therapy	54	Male	3	4	3	2	2	1
	70	Female	3	3	2	3	3	3
	68	Male	3	3	2	1	3	2
	58	Male	3	2	3	2	3	3
